# *LDLR* gene polymorphism (rs688) affects susceptibility to cardiovascular disease in end-stage kidney disease patients

**DOI:** 10.1186/s12882-021-02532-6

**Published:** 2021-09-23

**Authors:** Monika Buraczynska, Jerry Jacob, Karolina Gwiazda-Tyndel, Andrzej Ksiazek

**Affiliations:** 1 Department of Nephrology, Medical University of Lublin, Jaczewskiego 8, 20-950 Lublin, Poland; 2 Hope Medical Institute, Newport News, Virginia, USA

**Keywords:** LDL receptor gene, End-stage kidney disease, rs688 polymorphism, Genotyping, Risk allele

## Abstract

**Background:**

The low-density lipoprotein receptor (LDLR) plays a significant role in maintaining the cellular cholesterol homeostasis. Mutations in the *LDLR* gene can lead to a significant rise in plasma LDL levels that may result in an increased risk of atherosclerosis and coronary heart disease. The purpose of this study was to assess the potential association of the *LDLR* rs688 polymorphism with cardiovascular disease (CVD) in patients with end-stage kidney disease (ESKD) undergoing hemodialysis.

**Methods:**

In this case-control study the polymorphism was genotyped by the allele specific PCR method in 800 patients with ESKD and 500 healthy controls. The genotype and allele distribution was compared in subgroups of patients with CVD (552) versus those without CVD (248).

**Results:**

A significant difference was observed in genotype distribution among ESKD patients and healthy controls. The frequencies of the T allele and TT genotype in ESKD group were significantly higher, with OR (95% CI) 2.2 (1.87–2.6), *p* <  0.0001 and 5.84 (3.94–8.65), *p* <  0.0001, respectively. In the he ESKD cohort the distribution of the rs688 was compared between CVD+ and CVD- subgroups. A strong association of the polymorphism with the CVD risk was observed in this analysis. The frequencies of the T allele and TT genotype were significantly higher in CVD+ subgroup, with OR (95% CI) 3.4 (2.71–4.26), *p* <  0.0001 and 13.2 (7.87–22.09), *p* <  0.0001, respectively. A multivariate logistic regression analysis was performed to estimate the association between rs688 T variant and risk of CVD. After adjustment for age, sex, BMI, hypertension and diabetes, both CT and TT genotypes were associated with an increased risk of developing CVD in the dominant, recessive and codominant models of inheritance. No significant differences in serum LDL cholesterol levels were found when compared between genotypes.

**Conclusions:**

The present study is the first to demonstrate the association of the *LDLR* gene polymorphism with increased susceptibility to cardiovascular disease in ESKD patients. This finding needs further investigation to confirm that *LDLR* rs688 might be a novel genetic risk factor with some prognostic capacity for CVD in ESKD patients.

## Background

Cardiovascular disease (CVD) is a common cause of death in maintenance dialysis patients. In the United States it accounts for about 45% of reported deaths in this patient population [1]. Dialysis patients have a 10 to 30-fold higher incidence of cardiovascular death compared to general population [1, 2]. This high prevalence of CVD and increased mortality rate in the population of dialysis patients can only in part be explained by traditional cardiovascular risk factors such as age, obesity, hypertension, hyperglycemia or hyperlipidemia [3, 4]. Although the candidate gene approach and genome-wide association studies have successfully identified CVD susceptibility genes, such studies in chronic kidney disease patients are limited [5, 6].

The low-density lipoprotein receptor (LDLR) is a cell surface glycoprotein involved in binding and uptake of plasma LDL particles from the blood circulation by receptor-mediated endocytosis. It significantly contributes to cellular cholesterol homeostasis [7]. Polymorphic variants in the *LDLR* gene can induce a significant increase in plasma LDL levels, associated with a higher risk of atherosclerosis and coronary heart disease [8]. There are numerous mutations of the *LDLR* gene described that influence exons, splicing sites and the promoter regions. Some of these variants have been reported to cause familial hypercholesterolemia [9]. LDLR proteins are encoded by the 45 kb long *LDLR* gene consisting of 18 exons and located on chromosome 19 (19p13) [10].

A single nucleotide polymorphism (SNP) rs688, located in *LDLR* exon 12, is linked with low-density lipoprotein cholesterol and coronary artery disease (CAD), independently on gender [11]. The TT genotype of rs688 has shown association with hyperlipidemia [12] and with higher total and LDL-cholesterol levels [13, 14]. This synonymous SNP disrupts a splicing enhancer, causing an alternative exon splicing, which can result in a shift in the reading frame and altered gene transcript [13].

In this preliminary retrospective study we aimed to assess the potential association of the *LDLR* rs688 polymorphism with a risk of cardiovascular disease in hemodialysis patients with end-stage kidney disease (ESKD).

## Methods

### Patients and controls

The case-control study population consisted of 800 unrelated, adult patients with end-stage kidney disease (ESKD). Genomic DNA for this retrospective study was isolated from subjects treated with hemodialysis at the University Hospital and dialysis center of Medical University of Lublin between 2006 and 2019. All patients were Caucasians. Chronic kidney disease was diagnosed according to KDOQI (Kidney Disease Outcomes Quality Initiative) definition. According to KDOQI, ESKD was defined as estimated glomerular filtration rate (eGFR) < 15 ml/min/1.73 m^2^ associated with clinical signs of uremic syndrome, requiring dialysis. ESKD patients with dialysis duration less than 6 months, with diagnosed primary or secondary immunodeficiencies, on immunosuppressive therapy, with current pregnancy, malignancy and active systemic infection were excluded from the study. This was done to ascertain that all included patients have well defined disease phenotype. Some of the mentioned comorbidities could represent confounding factors and potentially affect the results. Cardiovascular disease was diagnosed in 552 patients (69%). All patients had the CVD diagnosis already established at the time of DNA sample collection. Cardiovascular disease was diagnosed as one or the combination of several pathological states: congestive heart failure, left ventricular hypertrophy, angina pectoris, ischemic heart disease, myocardial infarction, ischemic cerebral stroke. Clinical manifestations of CVD were confirmed by appropriate biochemical, radiographic, echocardiographic and vascular diagnostic criteria. A total of 539 patients were hypertensive and receiving antihypertensive medications. They fulfilled the World Health Organization criteria for hypertension. Hypertension was defined as a systolic blood pressure ≥ 140 mmHg and diastolic blood pressure ≥ 90 mmHg and ongoing treatment with antihypertensive medications. A complete medical history, laboratory determinations and physical examination were reviewed for all patients.

Apparently healthy individuals (*n* = 500), randomly recruited mainly among Medical University of Lublin hospital staff and blood donors who underwent health examination, were enrolled as a control group. All had normal ECG and no clinical evidence of CVD. The control subjects had no past history of kidney disease and underwent regular health examination. Their serum creatinine levels were tested before enrollment. Those with a positive family history of renal or cardiovascular disease in first-degree relatives were excluded from the study. After a full explanation of the study, a written informed consent for participating in this study was obtained from all patients and controls. The research protocol of the study was approved by the Ethics Committee of the Medical University of Lublin. The investigation conforms to the principles of the Declaration of Helsinki.

### Determination of *LDLR* rs688 genotype

**Fig. 1:**
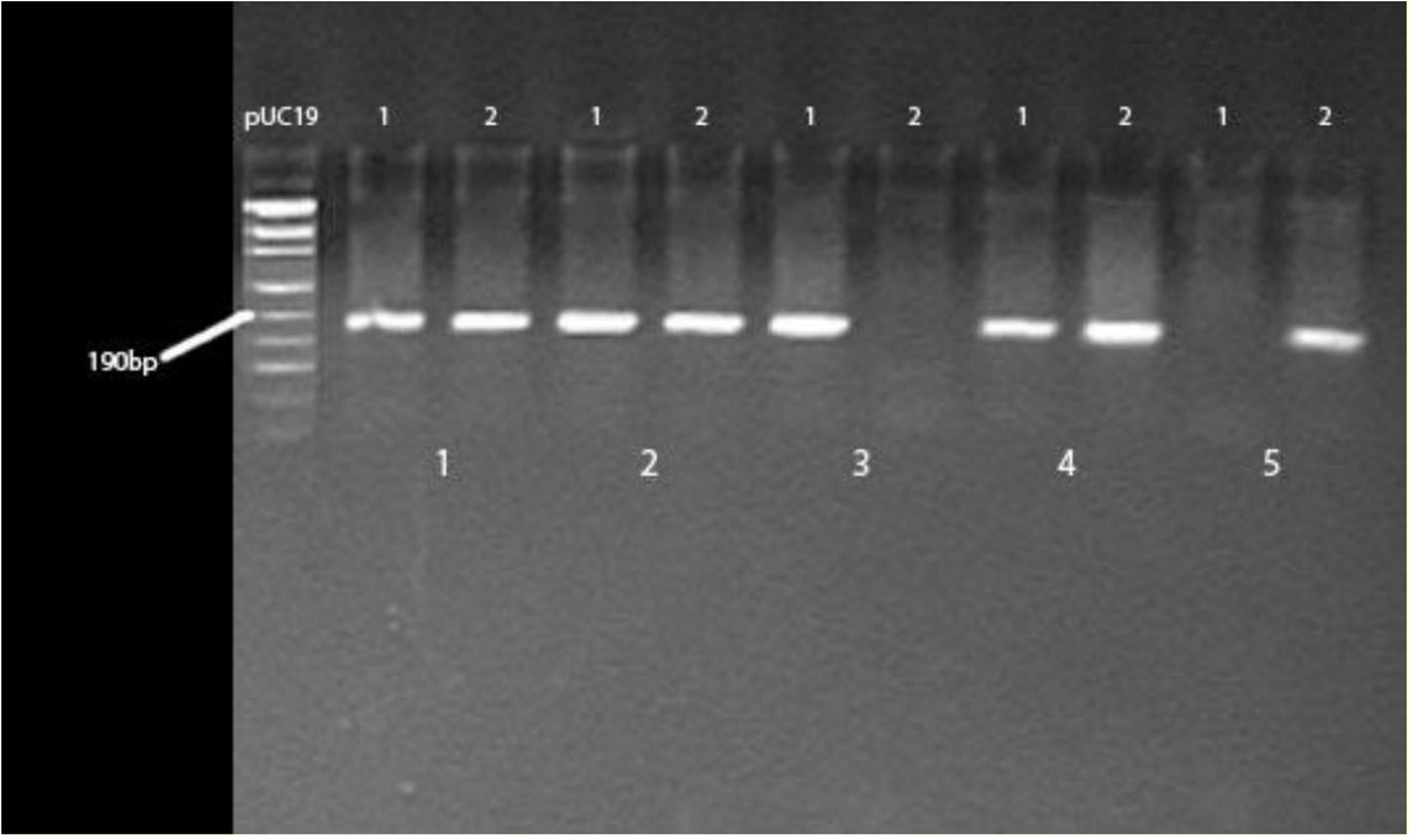
*LDLR* rs688 (C/T) polymorphism in the allele-specific PCR assay. A 191 bp DNA fragment indicates the presence of the allele in the sample. In each pair of reactions sample 1 represents C allele and sample 2 represents T allele. Legend: pUC19 DNA/Msp I (Hpa II) Marker. Genotypes: pair 1, 2, 4 - CT, pair 3 - CC, pair 5 - TT

Ten ml of peripheral whole blood were collected from each subject. Genomic DNA was isolated from leukocytes by the standard procedure. DNA concentration and purity of obtained samples were determined using Nano Drop 2000 (Thermo Scientific USA). DNA samples were stored at -70 °C before use. The rs688 polymorphism in the *LDLR* gene was detected through amplification of 191 bop DNA target by allele-specific polymerase chain reaction (PCR). The PCR reaction volume was 30 μl, containing 100 ng of genomic DNA, 10 x Taq buffer with KCL and 15 mM MgCl_2_, 200 mmol/l of each dNTP, 0.35 μl of forward primer and 0.30 μl of reverse primer and 2 U of Taq polymerase (all reagents from Thermo Scientific). For each DNA sample two reactions were set up, with F1 or F2 primer, each paired with reverse primer. The sequence of specific primers was: F1 (forward 1) primer 5′- CACTCCATCTCAAGCATCGATGTCAAC - 3′, F2 (forward 2) primer 5′- CACTCCATCTCAAGCATCGATGTCAAT - 3′ and reverse primer 5′- CAACCAGTTTTCTGCGTTCATCTTG − 3′ as reported earlier [12]. Slightly modified conditions for PCR reactions were applied: initial denaturation step at 95 °C for 5 min, followed by 35 cycles of denaturation at 95 °C for 30 s, annealing at 67 °C for 1 min and extension at 72 °C for 1 min. Amplification ended with a final extension step at 72 °C for 10 min. The resulting fragments were separated by electrophoresis on a 2% agarose gel with ethidium bromide. The length of the PCR product was 191 bp for both C and T allele (Fig. 1). Genotyping was blinded with respect to case-control status of the sample. The validation of genotyping was assessed by using double PCR reactions for 20% of random samples. Also, 20 randomly selected samples for each genotype were sequenced in CEQ 8000 Genetic Analysis System (Beckman Coulter, England) to confirm the correctness of genotype reading in agarose gel. A 100% concordance was observed between genotyping assays.

### Statistical analysis

Statistical calculations were executed using SPSS version 14.0 for Windows (SPSS, Inc., Chicago, IL. USA). For baseline descriptive data, the values of normally distributed variables are expressed as mean ± standard deviation (SD) or percentages as required. For comparisons of discrete and continuous variables between groups Student’s *t*-test and Mann-Whitney test were used. Potential deviation from Hardy-Weinberg balance was assessed using a chi-square goodness-of-fit test with 1 degree of freedom. The *LDLR* rs688 genotype distribution and allele frequencies in study groups were compared using a Pearson chi-square test of independence. For significant allelic and genotypic associations in different genetic models, the adjusted odds ratios (OR) with corresponding 95% confidence intervals (CI) were estimated. Non-risk allele or genotype were used as a reference. A multivariate logistic regression analysis was carried out to investigate the genotype impact and clinical profile associated with CVD risk in dialyzed patients and to verify the independence of the associations. ORs were adjusted for age, sex, BMI, hypertension and diabetes mellitus. Power estimations based on allele frequencies were done utilizing the program of Purcell et al. for case-control study, available at http://pngu.mgh.harvard.edu/~purcell/gpc/. For all two-sided tests the level of statistical significance of differences was set at *p* <  0.05.

## Results

A total of 800 hemodialyzed patients with ESKD were successfully genotyped with the rs688 polymorphism in the *LDLR* gene. There were 425 males with a mean age of 61.2 years and 375 females with a mean age of 64.3 years. The most frequent primary kidney diseases diagnosed in this group were: chronic glomerulonephritis (16%), diabetic nephropathy (27%) and interstitial nephritis (12%). A total of 552 patients were diagnosed with cardiovascular disease. In the control group of 500 healthy individuals there were 278 males (mean age 58.3) and 222 females (mean age 56.5). After validation of the genotyping procedure, there was a 100% concordance between genotypes obtained by PCR and those from sequencing. The comparison of clinical and laboratory characteristics of CVD+ and CVD-subgroups is presented in Table 1. No significant differences between CVD+ and CVD- subjects were observed in gender distribution, years on dialysis, total cholesterol and serum creatinine levels. Individuals in CVD+ subgroup showed greater prevalence of diabetes and hypertension (both *p* <  0.001).

**Table 1:** Demographic and clinical profile of ESKD patients according to the presence or absence of CVD

Variables	ESKD CVD+	ESKD CVD-	*p* value
N	552	248	
Gender (M/F)	306/246	119/129	0.053
Age at study (years)	67.4 ± 14.2	58.3 ± 15.3	< 0.001
Years on dialysis	4.8 ± 2.9	5.1 ± 3.6	0.210
Diabetes mellitus (%)	198 (36)	55 (22)	0.001
Hypertension (%)	420 (76)	119 (48)	< 0.001
BMI (kg/m^2^)	27.2 ± 5.3	25.9 ± 5.1	0.001
SBP (mmHg)	146 ± 9	143 ± 11	< 0.001
DBP (mmHg)	83 ± 7	81 ± 12	0.003
Total cholesterol (mmol/l)	4.7 ± 2.3	4.8 ± 1.7	0.539
HDL cholesterol (mmol/l)	1.2 ± 0.4	1.3 ± 0.5	0.002
Triglycerides (mmol/l)	1.8 ± 0.9	1.6 ± 0.9	0.004
Serum creatinine (μmol/l)	761 ± 147	782 ± 164	0.072

Values are presented as mean ± SD or numbers (%). ESKD, end-stage kidney disease. *CVD* Cardiovascular disease, *BMI* Body mass index. *SBP* Systolic blood pressure, *DBP* Diastolic blood pressure. Variable values determined by Student’s t-test for continuous and Mann Whitney test for discrete variables

The frequencies of the rs688 genotypes (CC, CT and TT) among ESKD patients and controls are presented in Table 2. There was a slight deviation from the Hardy-Weinberg equilibrium in the control group (χ^2^ = 4.219, *p* = 0.039) and no deviation in ESKD patient group (χ^2^ = 2.89, *p* = 0.089). The minor (T) allele frequency in the healthy population involved in this study was 32%. A statistically significant difference was observed in genotype and allele distribution among ESKD patients and healthy controls. In the ESKD group the frequencies of the T allele and TT genotype were significantly higher than in healthy subjects, with OR (95% CI) 2.2 (1.87–2.6), *p* <  0.0001 and 5.84 (3.94–8.65), *p* <  0.0001, respectively.

**Table 2:** Genotype and allele distribution of rs688 polymorphism in the *LDLR* gene in ESKD patients and controls

	Genotypes				MAF	OR (95% CI)	
	N	CC	CT	TT		T allele	TT genotype^a^
ESKD patients	800	184 (23)	424 (53)	192 (24)	0.50	2.2 (1.87–2.6) *p* < 0.001	5.84 (3.94–8.65) *p* < 0.001
Controls	500	224 (45)	236 (47)	40 (8)	0.32	ref.	ref.

ESKD, end-stage kidney disease.. LDLR, low density lipoprotein receptor. MAF, minor allele frequency. Genotype distribution is shown as numbers (%). Hardy-Weinberg equilibrium: χ^2^ = 4.219, *p* = 0.039 for control group; χ^2^ = 2.890, *p* = 0.089 for ESRD patients. ^a^Calculated versus CC genotype

The ESKD patient cohort was analyzed according to the presence or absence of CVD and the distribution of the rs688 polymorphism was compared between the CVD+ and CVD- subgroups (Table 3). The significant differences were observed in this analysis. There was a strong association of the T allele and TT genotype with the presence of cardiovascular disease in ESKD patients. The frequencies of the T allele and TT genotype were significantly higher in the CVD+ subgroup, with OR (95% CI) 3.4 (2.71–4.26), *p* <  0.0001 and 13.2 (7.87–22.09), *p* <  0.0001, respectively. At the T allele frequency 0.30 in CVD- subgroup and 0.60 in CVD+ subgroup, the statistical power for this comparison was 100%.

**Table 3:** Genotype and allele distribution of rs688 polymorphism in the *LDLR* gene in subgroups of ESKD patients

	Genotypes				MAF	OR (95% CI)	
	N	CC	CT	TT		T allele	TT genotype^a^
ESKD CVD+	552	60 (11)	326 (59)	166 (30)	0.60	3.40 (2.71–4.26) p < 0.0001	13.2 (7.87–22.09) *p* < 0.0001
ESKD CVD-	248	124 (50)	98 (39)	26 (11)	0.30	ref.	ref.

*ESKD* End-stage kidney disease. *LDLR* Low density lipoprotein receptor. *CVD* Cardiovascular disease. *MAF* Minor allele frequency. Genotype distribution is shown as numbers (%). ^a^Calculated versus CC genotype

A multivariate logistic regression analysis was performed to estimate the association between rs688 T variant and risk of CVD. Table 4 shows distribution of the *LDLR* rs688 polymorphism in CVD+ and CVD- patient subgroups, according to dominant, recessive and codominant models of inheritance. After adjustment for age, sex, BMI, hypertension and diabetes, the T allele, in both CT and TT genotypes, was associated with an increased risk of developing CVD in all models of inheritance (Table 4).

**Table 4:** Distribution of the *LDLR* rs688 polymorphism according to the model of inheritance

*LDLR* rs688	CVD+ subgroup	CVD- subgroup	OR (95% CI)	*p* value
C / T genotypes	(*n* = 552)	(*n* = 248)		
Codominant model				
CC	60	124	ref.	–
CT	326	98	6.87 (4.69–10.07)^a^	< 0.0001
TT	166	26	13.19 (7.87–22.09)^a^	< 0.0001
Dominant model				
CC	60	124	ref.	–
CT + TT	492	124	8.2 (5.68–11.82)^a^	< 0.0001
Recessive model				
CC + CT	386	222	ref.	–
TT	166	26	3.67 (2.35–5.73)^b^	< 0.0001

*LDLR* Low density lipoprotein receptor. *CVD* Cardiovascular disease. Genotype distribution is shown as numbers (%). Odds ratio is referred to ^a^CC homozygote and ^b^CC + CT genotypes

In our analysis no statistically significant differences in serum LDL cholesterol levels were found when compared between genotypes (data not shown).

## Discussion

Cardiovascular disorders are one of the main complications in chronic kidney disease, but the causes of the excess of cardiovascular complications are not clear.

A high percentage of cardiovascular disease in the ESRD patient population suggests the presence of a large number of risk factors. In addition to the classical risk factors common for the general population, there are numerous non-traditional risk factors, associated with renal insufficiency and renal replacement therapy, like chronic inflammation or increased oxidative stress. All these factors are inadequate for a full understanding of increased risk for CVD.

The genetic background is an important element of multifactorial pathology of cardiovascular disease. Thus, analyzing new candidate genes in CVD, even those with moderate effect, can lead to discovery of yet unknown genetic risk factors.

At present, the literature on a role of *LDLR* gene polymorphisms in human diseases is sparse, but the association of these variants with atherosclerosis and cardiovascular disease is already documented in several studies. The genome-wide association studies identified common variations at *LDLR* locus, strongly associated with proatherogenic lipid profile and cardiovascular disease [15]. According to previous findings, a minor allele (T) of the rs688, a coding synonymous SNP in exon 12 of *LDLR,* was associated with increased plasma LDL cholesterol levels in several populations. It was found to decrease the efficiency of exon 12 splicing [13]. Both the T allele and TT genotype increase the risk of coronary artery disease [11, 12].

Among several factors contributing to atherosclerosis and cardiovascular disease in ESKD population is dyslipidemia. Serum cholesterol and LDL concentrations are usually within or below the normal range in hemodialysis ESKD patients. Elevation of these levels in patients on peritoneal dialysis may be in part caused by acquired LDL receptor deficiency. There are no reports published on the effects of *LDLR* gene polymorphisms on CVD in chronic kidney disease patients. To the best of our knowledge, this study is the first to assess the association of the *LDLR* rs688 polymorphism with cardiovascular disease in a population of end-stage kidney disease patients. We selected this variant because of its well documented associations with changes in lipid profile and cardiovascular disease. The frequency of the risk allele (T) in our healthy control group was 32%. It was lower than 41% (*p* < 0.0001) in another European population. However, in that report individuals in control group had normal coronary arteries but had other cardiovascular disorders like valvular heart disease [11].

We observed a statistically significant difference in the rs688 distribution between ESKD patients and healthy controls. In the ESKD group the frequencies of the T allele and TT genotype were significantly higher, with OR (95% CI) 2.2 (1.87–2.6) and 5.84 (3.94–8.65), respectively. We compared a distribution of the rs688 polymorphism between CVD+ and CVD- individuals within the ESKD group. The results indicated that there is a strong association of the T allele and TT genotype with the presence of cardiovascular disease, with the frequencies of the T allele and TT genotype significantly higher in CVD+ subgroup, with OR (95% CI) 3.4 (2.71–4.26) and 13.2 (7.87–22.09), respectively. The study was sufficiently powered for this analysis. This result is in agreement with another European study, that compared the distribution of *LDLR* rs688 polymorphism in 692 CAD patients and 291 CAD free individuals. The carriers of rs688 T allele were more frequent among CAD patients and the T allele remained an independent risk of CAD after adjustment for all traditional risk factors including lipid profile [11]. There are also other studies reporting the association of *LDLR* gene polymorphisms with cardiovascular disorders. In an Indian study of 200 patients with coronary artery disease and 200 healthy controls, the TT genotype and T allele of rs688 were associated with an increased susceptibility to CAD. The authors observed that over 3- and 0.74-fold increase of risk of developing CAD were associated with TT genotype and T allele in studied population. They concluded that *LDLR* rs688 gene variant can be used as a predisposing genetic marker for coronary artery disease [12]. Another study from India reported the association of two other LDLR gene polymorphisms (rs5925 and rs1529729) with susceptibility to coronary artery disease [15].

Several studies have investigated whether common polymorphisms in the *LDLR* gene contribute to individual variations in serum lipid concentrations. Although, in a previous study the *LDLR* rs688 polymorphism was associated with plasma lipids [16], we did not find any significant association of this polymorphism with lipid variables in our studied population. This is in agreement with a study of Martinelli et al. [11]. The Mexican study analyzed the association between ApoE isoforms and the SNP rs688 in the *LDLR* gene with CVD risk factors in women. An association was observed between ApoE4 isoform with TT or CT genotypes of the rs688 SNP and high levels of LDL-cholesterol. However, this effect was the result of ApoA4 isoform presence, since no association was found between rs688 alone and LDL-cholesterol level [17]. This suggests that *LDLR* polymorphism may be associated with cardiovascular disease beyond the lipoprotein metabolism pathway. Although the effect of any single polymorphism in common diseases is rather small, the rs688 polymorphism in the *LDLR* gene seems to be one of potential risk factors in development of cardiovascular disease in ESKD patients. An interaction with some other genes might affect the association leading to underestimation or overestimation of a role of polymorphism in determining the phenotype. Further studies are needed in this direction.

As most of the studies with the case-control design, this study has some limitations. Although our results demonstrated a significant association of the *LDLR* rs688 polymorphism with chronic kidney disease itself and with cardiovascular disease in ESKD group of patients, they should be interpreted with caution. There is no validation cohort available at this point. Since it is a retrospective case-control study, the data may be influenced by a selection bias that cannot be excluded. To limit this possibility, the consecutive patients were included. Also, although we tried to adjust for known confounding risk factors, some comorbidities present in the end-stage kidney disease patient population might still represent a confounding factor.

Those might involve the residual kidney function affecting a CVD development in ESKD patients but unfortunately we did not have enough data to analyze this factor in our study.

## Conclusion

The present study is the first to demonstrate the association of the *LDLR* gene polymorphism with increased susceptibility to cardiovascular disease in end-stage kidney disease patients. This finding should stimulate further investigation to confirm that *LDLR* rs688 polymorphism might be a novel genetic risk factor with some prognostic capacity for CVD in ESKD patients.

## Data Availability

The datasets used and analyzed during the current study are available from the corresponding author on reasonable request.

## Abbreviations

CVD: Cardiovascular disease
eGFR: Estimated glomerular filtration rate;
ESKD: End stage kidney disease
HDL: High density lipoprotein
HY: Hypertension
KDOQI: Kidney Disease Outcomes Quality Initiative
LDL: Low density lipoprotein
LDLR: Low density lipoprotein receptor
MAF: Minor allele frequency
PCR: Polymerase chain reaction
SNP: Single nucleotide polymorphism
SPSS: Statistical Package for Social Sciences
